# Effects of vaccination on COVID-19 infection symptoms in multiple sclerosis patients

**DOI:** 10.1016/j.ensci.2024.100511

**Published:** 2024-06-12

**Authors:** Parisa Sharifi, Nasim Rezaeimanesh, Amir Moradi, Abdorreza Naser Moghadasi

**Affiliations:** a Multiple Sclerosis Research Center, Neuroscience Institute, Tehran University of Medical Sciences, Tehran, Iran; b Atherosclerosis Research Center, Ahvaz Jundishapur University of Medical Sciences, Ahvaz, Iran

**Keywords:** Multiple sclerosis, COVID-19 infection, COVID-19 vaccination, COVID-19 symptoms, Respiratory symptoms, Headache

## Abstract

**Background:**

Patients with multiple sclerosis (MS) are at higher risk of having infections due to receiving disease modifying therapies. The current study was conducted among Iranian MS patients who had experienced at least one episode of COVID-19 infection in order to evaluate the effects of COVID-19 vaccination on symptoms of their infection. Data on demographic information, MS characteristics, COVID-19 infection details, and vaccination status were collected. Statistical analyses, were performed to evaluate the association between vaccination and symptoms of COVID-19 infection.

**Methods:**

This cross-sectional study was conducted on confirmed MS patients. Demographic data and COVID-19 related symptoms were gathered via an online questionnaire. Confirmation of patients' who declared to be vaccinated was checked by their COVID-19 vaccination card.

**Results:**

A total of 236 MS patients participated in the study. The majority were female (79.7%), with a mean age of 36.1 ± 7.9 years. Among the participants, 72.5% had received the COVID-19 vaccine before their first episode of COVID-19 infection. The analysis showed a significant difference in the incidence of respiratory symptoms (*P*-value: 0.01) and headache (P-value: 0.04) between vaccinated and non-vaccinated individuals. Logistic regression analysis revealed that vaccinated MS patients had lower odds of developing respiratory symptoms (OR:0.29, 95% CI: 0.16 to 0.53, *P*-value<0.001) or headache (OR: 0.50, 95% CI: 0.25 to 0.98, P-value: 0.04) during their next COVID-19 infection episode. Moreover, MS patients who were receiving immunosuppressive drugs were less likely to have respiratory symptoms (OR:0.35, 95% CI: 0.16 to 0.77, *P*-value:0.009) but not headache (OR: 0.69, 95% CI: 0.30 to 1.60, P-value: 0.39).

**Conclusion:**

COVID-19 vaccination can reduce the incidence of respiratory symptoms and headaches in MS patients during COVID-19 infection episodes. Additionally, patients who are receiving immunosuppressive drugs may benefit from COVID-19 vaccination.

## Highlights

• 236 patients with multiple sclerosis participated to assess the effects of COVID-19 vaccination on their symptoms.
• The study demonstrated a significant decrease in respiratory symptoms and headaches among vaccinated participants.
• MS patients on immunosuppressive drugs showed a reduced chance of respiratory symptoms during COVID-19 infections.
• This study emphasizes the importance of COVID-19 vaccination for MS patients, due to its potential to reduce symptoms.

## 1 Introduction

The severe acute respiratory syndrome coronavirus type-2 that produced the COVID-19 pandemic has had a major impact, leading to notable rates of morbidity and mortality [1]. Since its emergence in December 2019, the global impact has been profound, with over 767 million confirmed cases and approximately 6.9 million associated deaths [2]. The COVID-19 virus can cause mild to severe sickness, and symptoms can manifest within 2 to 14 days following exposure [3]. COVID-19 infection mostly manifests with cough, fever, headache, fatigue and gastrointestinal symptoms such as nausea [[4], [5], [6]]. In response to this crisis, there has been an unprecedented effort to develop vaccines, recognized as highly effective tools for reducing illness and mortality rates [7]. The COVID-19 vaccine offers direct protection to individuals who have been immunized against the virus, as well as providing indirect protection to the entire community by reducing viral transmission and limiting its spread [8].

Multiple Sclerosis (MS) is a chronic autoimmune illness defining by demyelination of the central nervous system, primarily affecting young individuals, particularly women [9]. It is estimated that approximately 2.8 million individuals worldwide are living with MS, with a global prevalence of 35.9 cases per 100,000 people [10]. Patients with MS face an elevated risk of contracting specific types of infections, including respiratory, viral, and bacterial infections of various kinds [[11], [12], [13]]. Furthermore, certain disease-modifying therapies (DMTs) have been associated with an increased susceptibility to infections in MS patients, as they can suppress or modify the immune system [14,15]. The primary cause of mortality for MS patients is infections and it can exacerbate their symptoms. Therefore, it is crucial to prioritize the vaccination of these patients to reduce the risk of infection and its adverse effects [12]. Additionally, it has been estimated that MS relapses associated with an infection can lead to more severe neurological damage compared to relapses unrelated to the infection [16]. Vaccine hesitancy is a significant concern among individuals with MS due to their regular use of medications that affect the immune system. There are concerns about the effectiveness of vaccines in this population, as well as potential adverse effects on the course of their disease [17].

The available information on the symptoms of COVID-19 infection in individuals who received the COVID-19 vaccine compared to those who did not is limited. Clinical trials and studies focused primarily on the vaccine's efficacy, safety, and immunogenicity rather than comparing infection symptoms among vaccinated and non-vaccinated individuals. Therefore, it is crucial to monitor and manage symptoms of COVID-19 infection in MS patients who received COVID-19 vaccination. In addition, the effectiveness of COVID-19 vaccination on MS patients receiving immunosuppressors is still questionable. Therefore, in the current study, we aimed to examine the effects of vaccination on symptoms of COVID-19 infection in MS patients.

## 2 Methods

### 2.1 Study design and participants

This cross-sectional study conducted at the Multiple Sclerosis Research Center, Tehran University of Medical Sciences, aimed to investigate various aspects of COVID-19 vaccination on individuals with MS. The study included Iranian MS patients who met specific inclusion criteria, such as having a definite diagnosis of MS based on the revised McDonald criteria (2017) [18], having at least one episode of COVID-19 infection confirmed by either performing PCR test on nasal swab samples or lung computerized tomography scan, being at least 18 years old and having the capacity to complete an online Google form link through social media. Given the potential for asymptomatic COVID-19 infection, we could not be sure whether the patients had only one episode of infection before including in the study or not; Therefore, we relied on their first confirmed episode of infection.

### 2.2 Ethical approval

The goals of the study were thoroughly explained to participants before the questionnaire was handed out, and it was made clear that participation was voluntary. The Tehran University of Medical Sciences Ethics Committee evaluated and approved the study protocol, designating it with the ethics code “IR.TUMS.NI.REC.1400.024.”

### 2.3 Data collection

The questionnaire was developed by a panel of expert neurologists. A pilot study on 20 participants was conducted to assess the face validity of questionnaire and modifying questions in case of ambiguity. The questionnaire validity including test-retest reliability was assessed by a statistician. An online survey in the style of a Google Form was used for data collecting age, gender, MS type, current use of DMT, and year of MS onset were all gathered using the questionnaire. Additionally, it gathered data on COVID-19-related variables, such as the time of infection initiation, the quantity of infections encountered, fever, respiratory symptoms (cough and dyspnea), alterations in taste and smell perception, exhaustion, nausea, and headaches, and immunization history. Participants were able to access and complete the questionnaire by sharing the URL to the Google Form on social media platforms devoted to MS patient communities. Moreover, Confirmation of patients' who declared to be vaccinated was checked by their COVID-19 vaccination card.

### 2.4 Statistical analysis

Standard deviation (SD) and mean were presented for quantitative data, whereas percentage and number were reported for qualitative factors in descriptive statistics. To compare COVID-19 symptoms in MS patients who were vaccinated against those who were not, a chi-square test was used. We used logistic regression analysis to assess the relationship between the immunization and COVID-19 infection-related headaches and respiratory symptoms. The software used for data analysis was IBM Corp.'s SPSS version 26 (Armonk, NY). A significance level of *P*-value<0.05 was considered statistically significant.

## 3 Results

In the study involving 236 multiple sclerosis (MS) patients, the mean ± SD age of participants was 36.1 ± 7.9 years, and the mean ± SD disease duration was 8.3 ± 5.9 years. The majority of participants were female (N:188, 79.7%), and the most commonly used medication for MS was rituximab (N:91, 38.6%). Relapsing-remitting MS (RRMS) was the most prevalent type of MS among the participants (N:154, 65.3%). At the time of their first COVID-19 infection, 171 individuals (72.5%) of the participants had received COVID-19 vaccination (Table 1).

**Table 1:** Baseline characteristics of study participants.

Variables		Value
Age (years)		36.1 ± 7.9
Disease duration (years)		8.37 ± 5.9
Gender	Female	188(79.7%)
	Male	48(20.3%)
COVID-19 vaccination coverage	Yes	171(72.5%)
	No	65(27.5%)
MS Disease type	RRMS	154(65.3%)
	PRMS	20(8.5%)
	SPMS	62(26.3%)
MS Medication	Rituximab	91(38.6%)
	Fingolimod	37(15.7%)
	Dimethyl fumarate	27(11.4%)
	Interferon beta-1a (intramuscular)	25(10.6%)
	Interferon beta-1a (subcutaneous)	12(5.1%)
	Ocrelizumab	11(4.7%)
	Glatiramer acetate	9(3.8%)
	Natalizumab	7(3%)
	Teriflunomide	4(1.7%)
	Interferon beta-1b	4(1.7%)
	Azathioprine	1(0.4%)
	Mitoxantrone	1(0.4%)
	Other medications	4(1.7%)
	None	3(1.3%)

Quantitative variables are presented as mean ± SD, whereas qualitative variables are reported as number (%). MS, Multiple sclerosis; RRMS, Relapsing-remitting multiple sclerosis; PRMS, Progressive-relapsing multiple sclerosis; SPMS, Secondry-progressive multiple sclerosis.

The analysis showed no significant differences between vaccinated and non-vaccinated MS patients in their first episode of COVID-19 infection with regards to fever (*P*-value: 0.47), olfactory symptoms (anosmia or dysosmia) (P-value: 0.94), fatigue (P-value: 0.39), changes in taste sense (ageusia or dysgeusia) (*P*-value: 0.74), and nausea (P-value: 0.79). However, there was a significant difference regarding the incidence of respiratory symptoms (P-value: <0.001) and headache (P-value: 0.04). (Table 2)

**Table 2:** Comparison of COVID-19 infection symptoms between vaccinated and non-vaccinated people with MS.

Symptoms		COVID-19 vaccination		P-value
		no	yes	
Fever	no	66 (28.1%)	28 (11.9%)	0.47
	yes	105 (44.7%)	36 (15.3%)	
Respiratory	no	47 (20%)	36 (15.3%)	<0.001
	yes	124 (52.8%)	28 (11.9%)	
Olfactory	no	97 (41.3%)	36 (15.3%)	0.94
	yes	74 (31.5%)	28 (11.9%)	
Fatigue	no	67 (28.5%)	29 (12.3%)	0.39
	yes	104 (44.3%)	35 (14.9%)	
Taste	no	116 (49.4%)	42 (17.9%)	0.74
	yes	55 (23.4%)	22 (9.4%)	
Nausea	no	144 (61.3%)	53 (22.6%)	0.79
	yes	27 (11.5%)	11 (4.7%)	
Headache	no	110 (46.8%)	50 (21.3%)	0.04
	yes	61 (26%)	14 (6%)	

Qualitative variables are reported as number (%); P-value is calculated using Chi-squared test.

**Table 3:** Association of COVID-19 vaccination with respiratory symptoms and headache among total study participants and who were receiving immunosuppressors during their first episode of COVID-19 infection.

Symptoms		Respiratory symptoms				Headache			
		OR	95% CI		P-value	OR	95% CI		P-value
			Lower	Upper			Lower	upper	
Total study participants	Model 1	0.29	0.16	0.53	<0.001	0.50	0.25	0.98	0.04
	Model 2	0.26	0.13	0.49	<0.001	0.42	0.20	0.86	0.01
Study participants who were receiving immunosuppressors	Model 1	0.35	0.16	0.77	0.009	0.69	0.30	1.60	0.39
	Model 2	0.31	0.13	0.74	0.008	0.45	0.17	1.17	0.1

OR, odds ratio; CI, confidence interval.

Mode 1: Unadjusted regression model.

Model 2: regression model adjusted for age, gender, COVID-19 infection severity and MS type.

Logistic regression analysis was performed to further evaluate the effects of COVID-19 vaccination on the odds of patients having headaches or developing respiratory symptoms in COVID-19 infection episode. It was revealed that patients who had received the COVID-19 vaccine had lower odds of developing respiratory symptoms (OR: 0.29, 95% CI: 016 to 0.53, *P*-value<0.001). After adjustment for age, gender, MS disease type, and COVID-19 severity, we observed that the odds of developing respiratory symptoms in patients who had received COVID-19 vaccine was 74% lower compared with those who were not vaccinated (95% CI: 0.13 to 0.49, *P*-value<0.001). (Table 3).

Another observation was that MS patients who received the COVID-19 vaccine were 49.5% less likely to experience a headache in their COVID-19 infection episode compared to those who were not vaccinated (OR: 0.50, 95% CI: 0.25 to 0.98, *P*-value: 0.04). After adjusting for age, gender, MS disease type, and COVID-19 infection severity, the model showed that vaccinated participants had a 58% lower likelihood of developing a headache during their COVID-19 infection episode (OR: 0.42, 95% CI: 0.20 to 0.86, *P*-value: 0.01). (Table 3).

In the subgroup analysis of patients receiving immunosuppressing agents (rituximab, ocrelizumab, and fingolimod), a significant reduction was observed in patients who were vaccinated regarding respiratory symptoms (OR: 0.35, 95% CI: 0.16 to 0.77, *P*-value: 0.009). Additionally, after adjusting the model for age, gender, MS disease type, and COVID-19 infection severity, vaccinated participants still had significantly lower odds of developing respiratory symptoms. (OR: 0.31, 95% CI: 0.13 to 0.74, *P*-value: 0.008). (Table 3).

Moreover, vaccinated patients had a lower chance of developing headaches compared to non-vaccinated participants, although the difference was not statistically significant (OR: 0.69, 95% CI: 0.30 to 1.60, P-value: 0.39, respectively). After adjusting for age, gender, MS disease type, and COVID-19 infection severity, we observed that vaccinated participants had a lower chance of developing a headache during their COVID-19 infection episode, but the difference is still statistically insignificant. (OR: 0.45, 95% CI: 0.17 to 1.17, *P*-value: 0.10). (Table 3).

## 4 Discussion

Despite the extensive use of COVID-19 vaccines globally and numerous studies examining their safety and effectiveness in different groups, there is little information regarding the symptoms of COVID-19 infection in those who have received the vaccine. In the current study, we observed that MS patients who received the COVID-19 vaccine had a significantly reduced chance of experiencing respiratory symptoms and headaches in their first episode of COVID-19 infection compared with non-vaccinated participants. Moreover, we observed that vaccinated MS patients who were receiving immunosuppressive medications had significantly lower odds of developing respiratory symptoms compared with non-vaccinated MS patients.

COVID-19 infection can increase systematic inflammatory response and vascular damage, and it is associated with exacerbation of MS [19,20]. While having MS does not weaken the immune system, some DMTs used to treat MS can affect the immune system's ability to fight infections and suppress parts of the immune system, and some patients may experience long-term effects of the infection [[21], [22], [23]]. In a study by Gad et al. in Egypt it has been shown the frequency and severity of COVID-19 infection in MS patients were similar to general population and the risk of relapse in non-immunized MS patients after COVID-19 infection were higher than those who were vaccinated. [24] Therefore, it is important to prioritize MS patients for receiving the COVID-19 vaccine. MS patients are eligible to receive any COVID-19 vaccination, with the exception of live attenuated vaccines in those on immunosuppressive or immunomodulatory regimens [25]. However, it is important to address concerns regarding the efficacy of COVID-19 vaccines in the MS population, given that a considerable number of MS patients rely on immunosuppressive medications to manage their neurological symptoms [[26], [27], [28], [29], [30]].

Respiratory symptoms of COVID-19 infection can be various, such as cough, shortness of breath and dyspnea [4]. Numerous studies have demonstrated the effectiveness of COVID-19 vaccination in mitigating severe symptoms, hospitalization resulting from COVID-19 infection, mortality and long-COVID symptoms, including respiratory symptoms and headaches in the general population [[31], [32], [33], [34], [35], [36], [37], [38]]. According to a study by Taylor et al. Vaccines against COVID-19 could both prevent and help in treating long COVID [39]. The most typical signs of long COVID-19 are dyspnea and exhaustion, which can persist for months following acute COVID-19 [40]. Another study by Vanichkachorn et al. revealed that COVID-19 vaccination may not only lower a person's risk of developing long COVID, but it may also lessen the symptoms experienced by those who do, like chest pain, dizziness, and shortness of breath [41].

Neurological symptoms of COVID-19 infection are generally mild, with headache, anosmia, cerebrovascular disease and seizures being the most frequently reported [42]. The COVID-19 pandemic had an overall negative impact on patients with migraines, and COVID-19-infected patients with a previous history of migraines or headaches reported more severe headache attacks [[43], [44], [45]]. In addition, headache has been reported by a significant number of MS patients who have had COVID-19 [4,6]. Furthermore, headache is one of the COVID-19 infection symptoms that can reduce the quality of life in MS patients [46,47]. Therefore, it is crucial to monitor and manage headache symptoms in MS patients who have COVID-19 infection to improve their quality of life.

We postulate that the current observation regarding the reduction in respiratory symptoms and headaches can be explained through various mechanisms. COVID-19 vaccination can enhance the immune system's response to COVID-19 infection by stimulating both humoral and cellular immune responses. This, in turn, can potentially reduce viral replication, leading to shorter infection duration and decreased severity [48]. Additionally, vaccinated individuals have been found to have lower viral loads compared to non-vaccinated individuals in subsequent episodes of infection, which may contribute to milder symptoms [49]. Furthermore, it has been reported that non-vaccinated individuals may have immune system dysregulation, leading to more severe symptoms of infection [[50], [51], [52]]. COVID-19 vaccination can help downregulate the immune response [51], potentially reducing cell damage and resulting in less severe symptoms, including respiratory symptoms and headaches.

Available data suggest that patients using anti-CD20 therapies exhibit reduced humoral responses and diminished vaccine efficacy [26,[53], [54], [55]]. A study by Smith et al. found that patients receiving rituximab or other disease-modifying therapies (DMTs) were more likely to be hospitalized than MS patients not receiving these treatments. The study included about 4000 patients with MS who had received the COVID-19 vaccine [56]. Furthermore, Tallantyre et al. found that using anti-CD20 monoclonal antibodies and fingolimod was associated with lower seroconversion rates in MS patients following COVID-19 vaccination [57]. A Meta-analysis by Wu et al. also reported that MS patients who received anti-CD20 in the last six months prior to the vaccination may have diminished humoral response, while cellular response seems unaffected [58]. In the current study, we observed that vaccinated patients receiving immunosuppressive medications, including rituximab, ocrelizumab, and fingolimod, had lower odds of experiencing respiratory symptoms or headaches. However, the observed effect regarding headache incidence was not statistically significant. The observed discrepancy might be due to the fact that our study participants received different numbers of vaccine doses at the time of the study (one to three doses) with different intervals between the vaccination and their last dose of anti-CD20 medication. There was a decreased incidence of COVID-19 hospitalization among MS patients who had COVID-19 vaccinations more than six months after the last rituximab infusion [56]. Additionally, In a study conducted by Nishikubo et al. on hematological patients, it was suggested that patients who had recently received anti-CD20 could benefit from a third booster dose of the COVID-19 vaccine [59]. Therefore, although patients receiving immunosuppressors might benefit from COVID-19 vaccination, determining the optimal timing for administering the COVID-19 vaccine in these patients should be carefully evaluated.

## 5 Conclusion

COVID-19 vaccination in MS patients is associated with a lower likelihood of experiencing respiratory symptoms and headaches during COVID-19 infection episodes. Additionally, MS patients receiving immunosuppressor drugs benefit from COVID-19 vaccination regarding respiratory symptoms. Moreover, although vaccine efficacy in patients receiving immunosuppressors is still in question, it seems that these patients can benefit from the COVID-19 vaccine. Further studies are needed to determine the optimal vaccine administration timing in MS patients receiving immunosuppressive medications.

## CRediT authorship contribution statement

**Parisa Sharifi:** Writing – review & editing, Writing – original draft, Investigation, Formal analysis, Data curation, Conceptualization. **Nasim Rezaeimanesh:** Writing – review & editing, Validation, Resources, Methodology, Investigation, Data curation. **Amir Moradi:** Writing – review & editing, Writing – original draft, Investigation, Formal analysis. **Abdorreza Naser Moghadasi:** Writing – review & editing, Supervision, Resources, Methodology, Investigation, Formal analysis, Data curation, Conceptualization.

## Declaration of competing interest

The authors declare no competing interests regarding this article.
